# Eicosapentaenoic Acid Supplementation Changes Fatty Acid Composition and Corrects Endothelial Dysfunction in Hyperlipidemic Patients

**DOI:** 10.1155/2012/754181

**Published:** 2012-12-26

**Authors:** Ken Yamakawa, Michio Shimabukuro, Namio Higa, Tomohiro Asahi, Kageyuki Ohba, Osamu Arasaki, Moritake Higa, Yoshito Oshiro, Hisashi Yoshida, Tohru Higa, Taro Saito, Shinichiro Ueda, Hiroaki Masuzaki, Masataka Sata

**Affiliations:** ^1^Second Department of Internal Medicine, Endocrinology, Diabetes and Metabolism, Hematology and Rheumatology, University of the Ryukyus Graduate School of Medicine, Okinawa 903-0215, Japan; ^2^Diabetes and Life-Style Related Disease Center, Tomishiro Chuo Hospital, Okinawa 901-0243, Japan; ^3^Department of Cardio-Diabetes Medicine, The University of Tokushima Graduate School of Health Biosciences, 3-18-15 Kuramoto, Tokushima 770-8503, Japan; ^4^Cardiovascular Division, Naha City Hospital, Okinawa 902-8511, Japan; ^5^Cardiovascular Division, Tomishiro Chuo Hospital, Okinawa 901-0243, Japan; ^6^Cardiovascular Division, Shonan Hospital, Okinawa 904-0034, Japan; ^7^Heart Center, Wajiro Hospital, Fukuoka 811-0213, Japan; ^8^Department of Clinical Pharmacology and Therapeutics, University of the Ryukyus Graduate School of Medicine, Okinawa 903-0215, Japan; ^9^Department of Cardiovascular Medicine, The University of Tokushima Graduate School of Health Biosciences, 3-18-15 Kuramoto, Tokushima 770-8503, Japan

## Abstract

We investigated the effects of purified eicosapentaenoic acid (EPA) on vascular endothelial function and free fatty acid composition in Japanese hyperlipidemic subjects. In subjects with hyperlipidemia (total cholesterol ≥220 mg/dL and/or triglycerides ≥150 mg/dL), lipid profile and forearm blood flow (FBF) during reactive hyperemia were determined before and 3 months after supplementation with 1800 mg/day EPA. Peak FBF during reactive hyperemia was lower in the hyperlipidemic group than the normolipidemic group. EPA supplementation did not change serum levels of total, HDL, or LDL cholesterol, apolipoproteins, remnant-like particle (RLP) cholesterol, RLP triglycerides, or malondialdehyde-modified LDL cholesterol. EPA supplementation did not change total free fatty acid levels in serum, but changed the fatty acid composition, with increased EPA and decreased linoleic acid, **γ**-linolenic acid, and dihomo-**γ**-linolenic acid. EPA supplementation recovered peak FBF after 3 months. Peak FBF recovery was correlated positively with EPA and EPA/arachidonic acid levels and correlated inversely with dihomo-**γ**-linolenic acid. EPA supplementation restores endothelium-dependent vasodilatation in hyperlipidemic patients despite having no effect on serum cholesterol and triglyceride patterns. These results suggest that EPA supplementation may improve vascular function at least partly via changes in fatty acid composition.

## 1. Background

 Consumption of fish or fish oil is inversely correlated with morbidity and mortality due to cardiovascular disease [1–5]. The major components of fish oils are long-chain n-3 polyunsaturated fatty acids, eicosapentaenoic acid (EPA), and docosahexaenoic acid (DHA), which may have cardioprotective potential. Supplementation with purified EPA ethyl ester was shown to lower major coronary events in Japanese hypercholesterolemic patients (Japan EPA Lipid Intervention Study: JELIS) [6, 7]. Addition of 1800 mg/day EPA to low-dose statin treatment reduced the incidence of primary cardiovascular endpoints. In the JELIS study, the benefits of EPA were greater in patients with a prior history of coronary artery disease (CAD) (secondary prevention) [6] and in patients with multiple coronary risk factors [7]. Interestingly, such benefits were obtained without an effect of lowering low-density lipoprotein cholesterol (LDL-C) levels, but were more pronounced in populations consuming low amounts of n-3 fatty acids. However, the LDL-C-independent mechanism(s) of this phenomenon have not yet been clarified [6, 7].

 A variety of EPA actions such as antithrombotic [8, 9], lipid-lowering [10], anti-inflammatory [11, 12], and antiarrhythmic effects [13, 14] have been proposed as underlying mechanisms. Improvement of vascular endothelial function is one major candidate [15–17]. To assess vascular endothelial function, several modalities have been utilized including vasodilator response to endothelium-derived vasodilator factors such as acetylcholine and vasodilator response during reactive hyperemia determined by plethysmography or ultrasonic apparatus [18–22]. These methods revealed that purified EPA ethyl ester improved vascular endothelial function in high-risk patients with coronary artery disease [16] or hyperlipidemia [17].

 In the present study, we assessed the effects of purified EPA on vascular endothelial function in hyperlipidemic patients and sought comparable changes in lipid parameters including free fatty acid composition.

## 2. Methods

### 2.1. Subjects

Hyperlipidemic patients (*n* = 16) with hypercholesterolemia (total cholesterol ≥ 220 mg/dL) and/or hypertriglyceridemia (triglycerides ≥ 150 mg/dL) and age- and sex-matched normolipidemic healthy subjects (*n* = 18) were consecutively recruited from June 2000 to January 2002. Patients with a history of cardiovascular or cerebrovascular disease, hepatic or renal disease, diabetes mellitus, or heavy smoking were excluded. All hyperlipidemic patients were educated to maintain a low fat diet before and during the study period and then treated with a commercially available EPA supplement (Epadel capsule 300, Mochida Pharmaceutical Co. Ltd., Tokyo, Japan) containing EPA ethyl ester of >98% purity. Two 300 mg EPA capsules were administered 3 times per day orally after meals, for a total daily dose of 1800 mg. Before and 1 and 3 months after the start of EPA treatment, fasting blood samples were obtained, and vascular functions were determined as described later. During the study period, participants were directed not to change their regular diet and exercise habits, doses of regular medications were unchanged, and new prescriptions were avoided. The study protocols complied with the Guidelines of the Ethical Committee of the University of the Ryukyus, and the study was approved by the committee. Consent for participation was obtained from all subjects before the study.

### 2.2. Vascular Function

The study began at 8:30–9:30 AM after subjects fasted for at least 12 h. The subjects were kept in the supine position in a quiet, dark, air-conditioned room (constant temperature 22°C to 25°C) throughout the study. After 30 min in the supine position, basal forearm blood flow (FBF) was measured. Then the effect of reactive hyperemia and sublingual nitroglycerin (NTG) on FBF was measured. 

 FBF was measured using a mercury-filled silastic strain-gauge plethysmograph (EC-5R, D.E. Hokanson, Inc., Issaquah, WA) as previously described [20–22]. The strain gauge was attached to the right upper arm held above the right atrium and connected to a plethysmography device. A wrist cuff was inflated to 200 mmHg to exclude hand circulation from the measurements 1 min before and throughout each measurement of FBF. The upper arm cuff was inflated to 40 mmHg for 7 s in each 15 s cycle to occlude venous outflow from the arm using a rapid cuff inflator (EC-20, D.E. Hokanson, Inc.). FBF output signal was transmitted to a recorder (U-228, Advance Co., Nagoya, Japan). FBF was expressed as milliliters per minute per 100 mL of forearm tissue. An independent observer who had no knowledge of subjects' profiles calculated FBF. 

 To induce reactive hyperemia, FBF was occluded by inflating the cuff on the right upper arm to a pressure of 200 mmHg for 5 min. After release of the cuff, FBF was measured for 180 s. Nitroglycerin (NTG), 0.3 mg (Nihonkayaku Co., Tokyo, Japan), was then administered sublingually, and FBF was measured for 5 min. Peak FBF after brief episodes of hyperemia is almost exclusively mediated by nitric oxide (NO), reflecting vascular endothelial function [18, 19]. Meanwhile, an exogenous NO donor like a single dose of nitroglycerin (0.3 mg) is used to determine the maximum obtainable vasodilator response and to serve as a measure of endothelium-independent vasodilation, reflecting vascular smooth muscle function [18, 19]. In a preliminary study, after release of cuff or sublingual NTG, FBF returned to baseline within 10 min. Thus, end of the response to reactive hyperemia or sublingual NTG was followed by a 15 min recovery period. We confirmed the reproducibility of reactive hyperemia and sublingual NTG-induced vasodilation on 2 separate occasions in 28 healthy male subjects (mean age 27 ± 5 years) [20–22].

### 2.3. Blood Biochemical Measurements

 Venous blood samples were obtained in tubes containing EDTA sodium (1 mg/mL) and in polystyrene tubes without an anticoagulant. Plasma was immediately separated by centrifugation at 3,000 rpm at 4°C for 10 min, and serum was collected by centrifugation at 1,000 rpm at room temperature for 10 min. Samples were stored at 80°C until assayed. Routine chemical methods were used to determine serum concentrations of total cholesterol, HDL cholesterol, triglycerides, free fatty acids, creatinine, glucose, and electrolytes, and composition of nonesterified fatty acid was determined by gas chromatography. LDL-C concentration was estimated using Friedewald's method. Serum concentrations of cholesterol and triglycerides in remnant-like particles (RLP-C and RLP-TG) were assayed by an immunosorbent assay (Otsuka Pharmaceutical, Tokyo, Japan) as described [23]. Malondialdehyde-modified low-density lipoprotein (MDA-LDL), a form of oxidized LDL, was measured by enzyme-linked immunosorbent assay (SRL Inc., Tokyo, Japan). Levels of the inflammatory markers high-sensitivity C-reactive protein (hs-CRP) and serum amyloid A (SAA) were measured by a latex-enhanced nephelometric immunoassay (N Latex CRP II and N Latex SAA, Dade Behring Ltd., Tokyo, Japan). The homeostasis model of assessment-insulin resistance (HOMA-IR) was calculated using the following formula: fasting glucose (mg/dL) × fasting insulin (*μ*U/mL)/405. 

### 2.4. Statistical Analysis

 Values are expressed as the mean ± SD. Means were compared using 2-tailed unpaired Student's *t*-test or one-way analysis of variance (ANOVA). Comparisons of FBF time course curves during reactive hyperemia were analyzed by 2-way ANOVA for repeated measures on one factor followed by Bonferroni's correction for multiple-paired comparisons. The repeated factor was time of reactive hyperemia, and the nonrepeated factor was one group versus the other group. Multigroup comparisons of variables, including maximal FBF response to nitroglycerin, were performed using one-way ANOVA followed by Bonferroni's correction. Multiple logistic regression analysis was performed to adjust for confounding factors. Variables were treated as continuous, except for categorical class of hyperlipidemic and normolipidemic groups and sex, which were treated as nominal. Probabilities were considered to be significant if less than 0.05. The data were processed using Prism 5.0d (GraphPad Software, Inc., La Jolla, CA, USA) or JMP 9.0.3 software packages (SAS Institute Inc., Cary, NC, USA).

## 3. Results

### 3.1. Clinical Characteristics

 No patients withdrew from the study due to significant side effects. As shown in Table 1, sex and age were matched between the normolipidemic and hyperlipidemic groups. Subjects' diagnosis and current medications are listed in Table 1. Medication was not changed throughout the study period. As shown in Table 2, body weight, waist circumference, waist/hip ratio, and systolic blood pressure were higher in the hyperlipidemic group, but all of these variables were unchanged during the 3 months of EPA treatment.

### 3.2. Baseline Biochemical Profiles

 As shown in Table 3, fasting glucose, HbA1c, and HOMA-IR were higher in the hyperlipidemic group. With respect to lipid profile, total and LDL-C, triglycerides, Apo A2, B, C2, C3, and E were all higher in the hyperlipidemic group. RLP-C, RLP triglycerides, and MDA-LDL-C were also higher in the hyperlipidemic group. As shown in Table 4, palmitic (C16:0), palmitoleic (C16:1n-7), stearic (C18:0), oleic (C18:1n-9), linoleic (C18:2n-6), and dihomo-*γ*-linolenic (C20:3n-6) acids were all higher in the hyperlipidemic group than in normolipidemic group. The 2 groups did not significantly differ in myristic (C14:0), *γ*-linolenic (C18:3n-6), *α*-linolenic (C18:3n-3), arachidonic (AA C20:4n-6), eicosapentaenoic (EPA C20:5n-3), docosahexaenoic (DHA C22:6n-3) acids or EPA/AA levels. 

### 3.3. Baseline Endothelial Function

**Figure 1:**
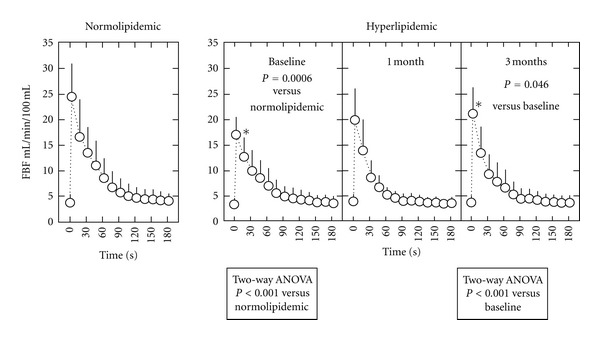
*Left panel*. Forearm blood flow (FBF) during reactive hyperemia in normolipidemic subjects (*n* = 18). *Right panel*. Effects of EPA on FBF during reactive hyperemia in hyperlipidemic subjects (*n* = 16). The *P* values for curve difference determined by 2-way ANOVA are shown. Data represent the mean ± SD. *P < 0.05 versus normolipidemic subjects and ^†^ *P* < 0.05 versus baseline.

 As shown in Figure 1, peak FBF during reactive hyperemia in the hyperlipidemic group (15.4 ± 6.1 mL/min/100 g) was less than that in the age- and sex-matched normolipidemic control group (22.8 ± 1.2, *P* < 0.01). The difference in peak FBF between the 2 groups was significant after adjustment for age, sex, and body mass index (BMI) (*P* = 0.025). However, the difference was lost after adjustment for those 3 factors in addition to LDL-C (*P* = 0.053), total free fatty acids (*P* = 0.068), palmitic acid (*P* = 0.853), or EPA/AA (*P* = 0.740). Maximal FBF after sublingual administration of NTG was similar in both groups (5.43 ± 0.64 mL/min/100 g versus 4.93 ± 0.36, not significant). 

### 3.4. Endothelial Function and Fatty Acid Composition

 As shown in Table 3, 3-month EPA supplementation did not change plasma levels of glucose, HbA1c, or HOMA-IR or serum levels of total, LDL-, or HDL-C, apolipoproteins, RLP-C, RLP triglycerides, or MDA-LDL-C in hyperlipidemic patients. EPA supplementation did not change levels of total free fatty acids (Table 3), but changed the composition of free fatty acids: EPA lowered linoleic (C18:2n-6), *γ*-linolenic (C18:3n-6), and dihomo-*γ*-linolenic (C20:3n-6) acids (Table 4) and most notably increased EPA by 3.1-fold and EPA/AA by 3.3-fold (see Supplementary Figure available online at doi:10.1155/2012/754181). EPA treatment tended to lower levels of SAA and hs-CRP, which were different between groups at baseline (Table 3).

**Figure 2:**
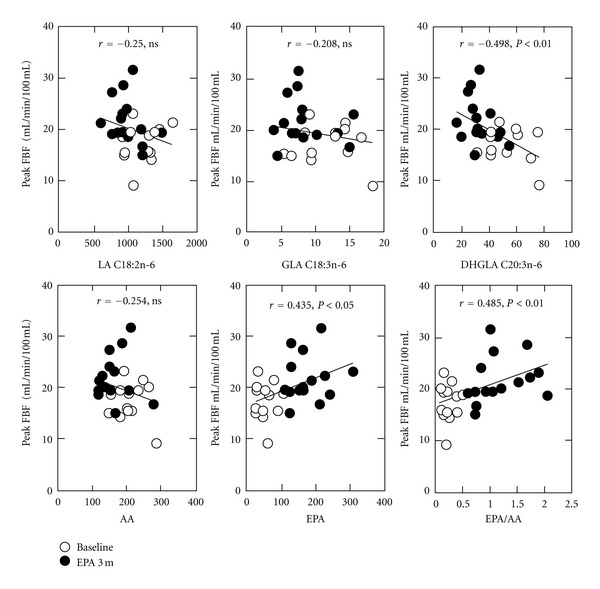
Correlation between peak forearm blood flow (peak FBF) and fatty acids in hyperlipidemic subjects. Peak FBF at baseline (open circles) and at 3 months after EPA supplementation (closed circles) is plotted to individual fatty acid values. Pearson's correlation coefficients (*r*) and *P* values (*P*) are shown. For abbreviations, see Table 4.

 After 3 months, EPA supplementation mildly but significantly increased the peak FBF (17.1 ± 3.5 versus 21.7 ± 4.4 mL/min/100 g, *P* < 0.001) of hyperlipidemic patients to a level comparable to normolipidemic controls (Figure 1, Supplementary Figure). Response to sublingual NTG did not change after EPA supplementation. Recovery of peak FBF was not correlated with changes in lipoproteins, apolipoproteins, RLP-C, RLP-triglycerides, or MDA-LDL-C. Recovery of peak FBF was not associated with total free fatty acid levels, but was correlated positively with EPA and EPA/AA and inversely with dihomo-*γ*-linolenic acid (C20:3n-6) (Table 4 and Figure 2).

## 4. Discussion

The major findings of the present study were, *first*, that 3-month supplementation with EPA recovered peak FBF during reactive hyperemia in hyperlipidemic subjects and, *second*, that recovery of peak FBF was not correlated with changes in lipid and lipoprotein profiles, but correlated positively with EPA and EPA/AA and inversely with dihomo-*γ*-linolenic acid (C20:3n-6).

### 4.1. Effects of EPA on Biochemical Profiles

 After 3-month supplementation, EPA did not change serum levels of total, LDL-C, or HDL-C or apolipoproteins in our hyperlipidemic patients. EPA has been reported to lower plasma triglyceride levels but demonstrates no definite effects on levels of total, LDL-C, or HDL-C [5–7, 24]. Although one study reported favorable effects of EPA on atherogenic triglyceride-rich lipoproteins and RLP-C [25], EPA treatment did not affect levels of triglycerides, RLP-C, RLP-triglycerides, or MDA-LDL-C in the present study. Previous EPA or fish-oil trials have demonstrated large interstudy variability in lipid parameters, which may be attributable to dose administered, intervention time, health status of participants, background diet, and many other confounding factors [5–7, 24]. Thus, our observation is not necessarily inconsistent with previous reports. By contrast, our finding is that EPA supplementation changed the composition of free fatty acids without changing total levels of free fatty acids in consistency with previous reports [5–7, 24, 25].

### 4.2. Baseline Endothelial Function

 Compared to the normolipidemic group, peak FBF during reactive hyperemia was largely decreased, and maximal FBF after sublingual administration of NTG was similar in the hyperlipidemic group. The difference in peak FBF between the 2 groups was lost after adjustment for age, sex, and BMI plus either LDL-C, total free fatty acids, palmitic acid, or EPA/AA, suggesting that free fatty acid, as well as LDL-C, could be related to endothelial dysfunction in hyperlipidemic patients.

### 4.3. Effects of EPA on Endothelial Function and Fatty Acid Composition

 Supplementation with EPA recovered the decrease in peak FBF during reactive hyperemia in hyperlipidemic patients to a comparable level to that of normolipidemic controls. The recovery in peak FBF was not correlated with changes in lipoproteins, apolipoproteins, RLP-C, RLP-triglycerides, or MDA-LDL-C. Recovery in peak FBF was not associated with total free fatty acid levels, but was correlated positively with EPA and EPA/AA and inversely with dihomo-*γ*-linolenic acid (C20:3n-6). In the JELIS study, the same amount of EPA reduced major coronary events in statin-treated hypercholesterolemic patients, despite no significant changes in LDL-C levels [6, 7]. However, the mechanism(s) of this effect beyond LDL-C were not determined. To the best of our knowledge, the present study is the first report to show that recovery of endothelial function was positively correlated with EPA and EPA/AA. 

 Endothelium-dependent function is a predictor of future cardiovascular events [18]. Thus, the improvement in endothelial function, as correlated with EPA and EPA/AA levels, may play a role in the reduction of cardiovascular events. Subanalysis of the JELIS study revealed that a higher plasma level of EPA at entry was inversely associated with the risk of major coronary events (hazard ratio = 0.71, *P* = 0.018, in the EPA intervention group) [24]. We previously reported that serum total free fatty acid levels were negatively correlated with peak FBF during hyperemia in subjects with no prior cardiovascular events (*r* = −0.277, *P* < 0.05) [20]. In the current study, EPA supplementation changed the composition of serum free fatty acids, with increases in EPA and EPA/AA and a decrease in dihomo-*γ*-linolenic acid (C20:3n-6). Recomposition of free fatty acids by EPA supplementation may avoid “unfavorable” fatty acid-induced endothelial dysfunction. 

 We reported saturated fatty acids as one candidate leading to endothelial dysfunction in a nondiabetic general population [20]. EPA supplementation possibly improves endothelium-dependent vascular function through an enhancement of NO release, in which saturated fatty acids such as palmitic acid can deteriorate [22, 26, 27]. Interestingly, a decrease in dihomo-*γ*-linolenic acid was correlated with improved endothelial function. However, whether levels of dihomo-*γ*-linolenic acid have an impact on cardiovascular events is unclear [28]. Nuts, seeds, and vegetable oils containing *α*-linolenic acid (ALA) are other dietary sources of n-3 fatty acids (plant n-3 fatty acids). Since a plant n-3-rich diet had been shown to improve endothelial function in hypercholesterolemic subjects [29], n-3 fatty acids, regardless of source, could have endothelium-protective mechanism(s).

### 4.4. Study Limitations

*First*, this study was observational, without a placebo group and in a limited number of patients. Various confounding factors can be included. Particularly, caution must be exercised in interpreting the effects of confounding diabetes mellitus, metabolic syndrome, smoking, and hypertension, which are all known to cause endothelial dysfunction. *Second*, patients took regular medication that potentially influenced endothelial function, such as calcium channel blockers, angiotensin converting enzyme inhibitors, and angiotensin receptor blockers. Although we were careful to ensure no change of medication doses or new prescriptions, the effects of regular medications could not be fully eliminated. *Third*, we performed noninvasive FBF measurement by strain-gauge plethysmography during reactive hyperemia to assess endothelial function because invasive measurements of FBF involve intra-arterial infusion of vasoactive agents. Vasodilation during reactive hyperemia is known to have multiple causes including metabolic factors other than nitric oxide. *Fourth*, our study does not verify a cause-and-effect process in the codevelopment of changes in fatty acid composition and improvement in vascular function. A confirmatory study to measure relationships between fatty acid composition and vascular function may be warranted.

## 5. Conclusion

 EPA supplementation restored endothelium-dependent vasodilator response in hyperlipidemic patients despite having no effect on serum cholesterol and triglyceride patterns. These results suggest that EPA supplementation may improve vascular function at least partly via changes in fatty acid composition and warrant investigation as an alternative strategic approach beyond LDL-cholesterol lowering.

## Supplementary Material

Supplementary Figure: Ratio of eicosapentaenoic acid to arachidonic acid (EPA/AA) (left panel) and peak forearm blood flow during reactive hyperemia (right panel) at baseline and at 3 months after EPA supplementation in hyperlipidemic subjects (*n* = 16).

## Figures and Tables

**Table 1:** Profiles of studied subjects.

Gender	Age (yr)	Diagnosis	Drugs
Normolipidemic			

F	46	APS	None
F	55	HT	Valsartan, 40 mg
F	60	GD	Thiamazole, 20 mg
F	60	GD	Thiamazole, 7.5 mg
F	62	HT	Amlodipine, 5 mg
F	64	Osteoporosis	Alfacalcidol, 0.5 *μ*g; calcium carbonate 1.0 g
F	65	IHA	Spironolactone, 75 mg
F	68	None	None
F	68	VSA	Diltiazem-R, 100 mg
F	73	None	None
F	77	GD	Thiamazole, 30 mg; propranolol, 30 mg1
M	41	HT	Candesartan, 8 mg
M	43	IDA	Dried ferrous sulfate, 210 mg
M	44	GD	Propylthiouracil, 200 mg
M	56	GD, PUD	Thiamazole, 20 mg; lansoprazole, 15 mg; sucralfate, 3.0 g
M	66	None	None
M	68	HT	Amlodipine, 5 mg
M	76	None	None

mean	61 ± 11		

Hyperlipidemic			

F	51	HL	None
F	56	HL, HT	Valsartan, 80 mg
F	63	HL	None
F	63	HL	None
F	64	HL	None
F	65	HL, HT	Amlodipine, 5 mg
F	71	HL, HT, T2DM, CSA	Losartan, 50 mg; benidipine, 4 mg; metformin, 750 mg; ticlopidine, 200 mg; nicorandil, 15 mg
F	73	HL, HT	Manidipine, 40 mg
F	74	HL	None
F	75	HL, HT, T2DM	Amlodipine, 5 mg; losartan, 50 mg; gliclazide, 160 mg
M	43	HL	None
M	48	HL, HT	Candesartan, 12 mg
M	51	HL, gout	Atorvastatin, 10 mg; allopurinol, 100 mg
M	66	HL, HT, VSA, gout	Losartan, 50 mg; diltiazem-R, 100 mg; allopurinol, 100 mg
M	68	HL, HT, PS	Amlodipine, 5 mg; enalapril, 5 mg; amantadine, 150 mg; levodopa, 200 mg
M	73	HL, HT, PUD	Barnidipine, 10 mg; prazosin, 0.25 mg; lansoprazole, 15 mg

mean	62 ± 12		

APS: anti-phospholipid antibody syndrome; HT: hypertension; GD: Graves' disease; IHA: idiopathic hyperaldosteronism; VSA: vasospastic angina; IDA: iron deficiency anemia; PUD: peptic ulcer disease; HL: hyperlipidemia; T2DM: type 2 diabetes mellitus; CSA: chronic stable angina; PS: Parkinson's syndrome; PUD: peptic ulcer disease; R: the retarded form of each drug.

**Table 2:** General characteristics of studied patients.

	Normolipidemic (*n* = 18)	Hyperlipidemic (*n* = 16)		
		0M	1M	3M
Body weight, kg	56.1 ± 11.1	61.7 ± 12.0	62.6 ± 12.9	61.0 ± 11.4
Body mass index, kg/m^2^	23.0 ± 3.3	26.3 ± 3.4*	26.8 ± 3.2*	25.0 ± 2.5
Waist circumference, cm	83.7 ± 9.1	91.4 ± 6.0*	95.4 ± 5.4*	92.0 ± 4.6*
Hip circumference, cm	93.2 ± 4.2	93.3 ± 5.1	96.3 ± 5.7	91.6 ± 4.8
Waist/Hip	0.90 ± 0.08	0.98 ± 0.05*	0.98 ± 0.09*	1.01 ± 0.06*
Systolic blood pressure, mmHg	120 ± 13	135 ± 20*	132 ± 21	131 ± 12
Diastolic blood pressure, mmHg	69 ± 9	75 ± 11	77 ± 15	77 ± 9
Heart rate, beats/min	64 ± 12	69 ± 14	77 ± 22	59 ± 12

Mean ± SD, *P < 0.05 versus normolipidemic.

0M, 1M, and 3M: baseline and 1 and 3 months after EPA treatment.

**Table 3:** Effects of EPA on blood biochemical parameters.

	Normolipidemic (*n* = 18)	Hyperlipidemic (*n* = 16)		
		0M	1M	3M
Glucose, mg/dL	92 ± 10	121 ± 32**	115 ± 32**	114 ± 26**
Insulin, *μ*U/mL	6.65 ± 4.74	8.59 ± 3.89	10.34 ± 5.29	6.64 ± 2.93
HbA1c, % (NGSP)	5.49 ± 0.34	6.90 ± 1.48**	6.67 ± 1.40**	6.90 ± 1.70**
HOMA-IR	1.55 ± 1.19	2.78 ± 1.88*	3.19 ± 2.34*	2.21 ± 1.36
HOMA-B	82 ± 48	60 ± 26	78 ± 31	56 ± 30
Total cholesterol, mg/dL	178 ± 24	243 ± 29***	244 ± 26***	232 ± 32***
Triglycerides, mg/dL	95 ± 47	209 ± 92***	204 ± 144***	216 ± 138***
HDL cholesterol, mg/dL	55 ± 15	53 ± 13	58 ± 13	52 ± 13
LDL cholesterol, mg/dL	105 ± 21	148 ± 35***	156 ± 31***	145 ± 40***
Free fatty acids, mmol/L	0.67 ± 0.29	0.68 ± 0.24	0.68 ± 0.16	0.59 ± 0.16
ApoA1, mg/dL	137 ± 21	142 ± 27	149 ± 24	143 ± 22
ApoA2, mg/dL	24 ± 5	29 ± 5*	29 ± 5*	29 ± 5*
ApoB, mg/dL	85 ± 10	121 ± 16***	130 ± 21***	125 ± 26***
ApoC2, mg/dL	2.00 ± 1.66	9.09 ± 5.86**	6.08 ± 2.97**	8.35 ± 4.50**
ApoC3, mg/dL	7.08 ± 1.68	12.53 ± 5.60**	13.37 ± 6.19**	13.99 ± 6.05**
ApoE, mg/dL	3.87 ± 1.09	6.20 ± 1.95***	6.75 ± 2.54***	6.72 ± 2.25***
A1/B	1.63 ± 0.33	1.21 ± 0.33	1.93 ± 2.78	1.80 ± 2.49
RLP cholesterol, mg/dL	4.1 ± 3.3	7.5 ± 3.2*	7.0 ± 3.6*	7.4 ± 4.0*
RLP triglycerides, mg/dL	16 ± 14	36 ± 20*	32 ± 28	32 ± 29
MDA-LDL cholesterol, mg/dL	94 ± 21	165 ± 53***	175 ± 57***	152 ± 47***
White blood cells, /*μ*L	6157 ± 1509	6880 ± 2932	6513 ± 2600	6455 ± 2314
Fibrinogen, mg/dL	298 ± 53	372 ± 64*	386 ± 96*	362 ± 31
Serum amyloid A (SAA), mg/dL	3.00 ± 0.78	11.44 ± 10.93*	6.19 ± 6.06	5.65 ± 3.29
High-sensitive C-reactive protein, *μ*g/mL	531 ± 441	4912 ± 4638*	2215 ± 3326	2795 ± 3247

Mean ± SD, *P < 0.05, **P < 0.01, ***P < 0.001 versus normolipidemic. 0M, 1M, and 3M: baseline and 1 and 3 months after EPA treatment.

**Table 4:** Effects of EPA on blood fatty acids composition.

		Normolipidemic (*n* = 18)	Hyperlipidemic (*n* = 16)		
			0M	1M	3M
Myristic, C14:0	*μ*g/mL	21 ± 12	31 ± 11	33 ± 19	32 ± 19
Palmitic, C16:0	*μ*g/mL	668 ± 156	989 ± 265*	986 ± 404*	913 ± 390
Palmitoleic, C16:1n-7	*μ*g/mL	52 ± 15	82 ± 38*	88 ± 52	79 ± 49
Stearic, C18:0	*μ*g/mL	221 ± 41	272 ± 66*	273 ± 70*	265 ± 68
Oleic, C18:1n-9	*μ*g/mL	617 ± 177	907 ± 249**	856 ± 439	783 ± 410
Linoleic, LA C18:2n-6	*μ*g/mL	935 ± 200	1203 ± 228**	1060 ± 248	996 ± 222^†^
*γ*-Linolenic, GLA C18:3n-6	*μ*g/mL	8.3 ± 5.6	11.7 ± 4.0	8.8 ± 2.8	8.3 ± 3.7^†^
*α*-Linolenic acid, ALA C18:3n-3	*μ*g/mL	27 ± 21	38 ± 15	37 ± 14	37 ± 15
Dihomo-*γ*-linolenic acid, DHGLA C20:3n-6	*μ*g/mL	34 ± 10	52 ± 15**	35 ± 13^††^	32 ± 10^†††^
Arachidonic acid, AA C20:4n-6	*μ*g/mL	182 ± 41	209 ± 40	197 ± 45	164 ± 45^†^
Eicosapentaenoic, EPA C20:5n-3	*μ*g/mL	45 ± 26	52 ± 26	161 ± 49^∗∗∗†††^	180 ± 55^∗∗∗†††^
Docosahexaenoic, DHA C22:6n-3	*μ*g/mL	122 ± 46	159 ± 60	162 ± 68	157 ± 65
EPA/AA		0.24 ± 0.12	0.25 ± 0.12	0.84 ± 0.29^∗∗∗†††^	1.16 ± 0.46^∗∗∗†††^

Mean ± SD, *P < 0.05, **P < 0.01, ***P < 0.001 versus normolipidemic, ^†^ *P* < 0.05, ^†††^ *P* < 0.001 versus 0M. 0M, 1M, and 3M: baseline and 1 and 3 months after EPA treatment.

## Abbreviations

AA: Arachidonic acid
ANOVA: One-way analysis of variance
C: Cholesterol
CAD: Coronary artery disease
DHA: Docosahexaenoic acid
EPA: Eicosapentaenoic acid
FBF: Forearm blood flow
HDL: High-density lipoprotein
HOMA-IR: Homeostasis model of assessment-insulin resistance
hs-CRP: High-sensitivity C-reactive protein
JELIS: Japan EPA Lipid Intervention Study
LDL: Low-density lipoprotein
MDA-LDL: Malondialdehyde-modified low-density lipoprotein
NTG: Nitroglycerin
RLP: Remnant-like particle
SAA: Serum amyloid A
VLDL: Very low density lipoprotein.
