# Supplementary material for: Eicosapentaenoic Acid Supplementation Changes Fatty Acid Composition and Corrects Endothelial Dysfunction in Hyperlipidemic Patients
Source: Cardiol Res Pract. 2012 Dec 26;2012:754181. doi: 10.1155/2012/754181 (PMC3541561; doi:10.1155/2012/754181)
Supplement: Supplementary file 1 — Supplementary Figure: Ratio of eicosapentaenoic acid to arachidonic acid (EPA/AA) (left panel) and peak forearm blood flow during reactive hyperemia (right panel) at baseline and at 3 months after EPA supplementation in hyperlipidemic subjects (n = 16). [file 754181.f1.pdf]

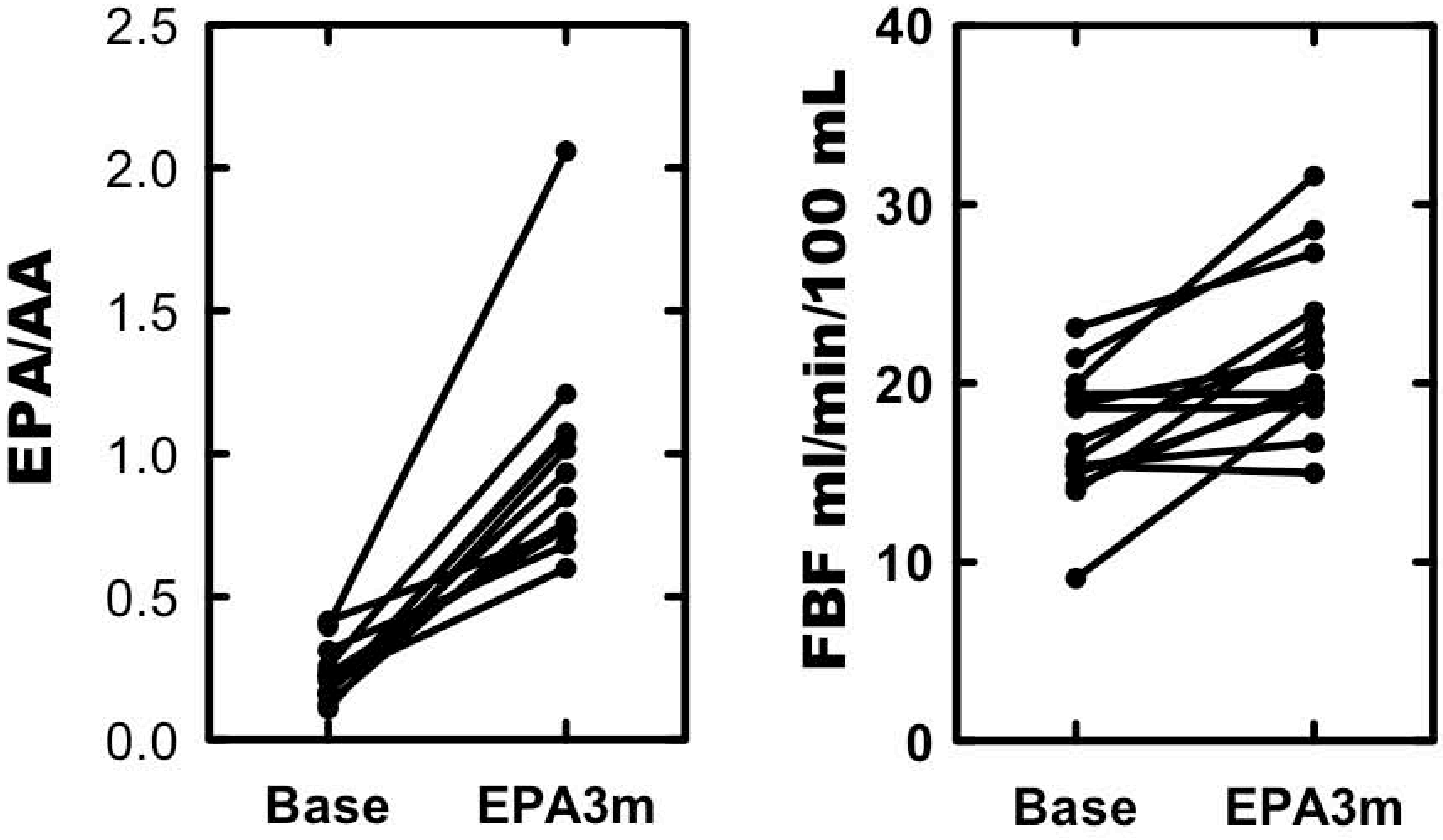

Supplementary Fig. Ratio of eicosapentaenoic acid to arachidonic acid (EPA/AA) (**left panel**) and peak forearm blood flow during reactive hyperemia (**right panel**) at baseline and at 3 months after EPA supplementation in hyperlipidemic subjects (n = 16)
